# Assessing the Effect of a Rehabilitation Intervention on Exhaustion Disorders, Depression, and Return to Work: A Randomized Controlled Trial

**DOI:** 10.1007/s10926-025-10350-y

**Published:** 2025-12-13

**Authors:** Emelie Thunqvist, Åke Nygren, Björn Löfman, David Parker, Emma Brulin

**Affiliations:** 1 Unit of Occupational Medicine, Institute of Environmental Medicine, Karolinska Institute, Stockholm, Sweden; 2 Center for Occupational and Environmental Medicine, Stockholm Region, Stockholm, Sweden; 3 Department of Clinical Sciences, Danderyd Hospital, Karolinska Institute, Stockholm, Sweden; 4 The Back Institute, Pain and Stress Rehabilitation, Stockholm, Sweden

**Keywords:** Stress-related ill-health, Exhaustion disorder, Burnout, Depression, Rehabilitation, Health care professionals, RCT

## Abstract

**Purpose:**

The aim was to evaluate the effect of a Rehabilitation to Work (ReTA) model on symptoms of exhaustion disorder (ED), depression, and return to work (RTW) among healthcare workers on long-term sickness absence (LTSA) for stress-related ill-health.

**Methods:**

The ReTA was set up as a randomized control trial (RCT), including 30 in the intervention and 41 in the control group. Physicians and nurses on LTSA were recruited via a national insurance database. The degree of self-reported ED and depression was measured using the Karolinska Exhaustion Disorder Scale (KEDS), and the Montgomery Åsberg Depression Rating Scale (MADRS). Data was collected at baseline, 3-month, and 1-year follow-up. A linear mixed model and Pearson chi-square were used to examine between- and within-group differences in KEDS, MADRS, and RTW.

**Results:**

At baseline, no differences in KEDS and MADRS were observed between the groups. KEDS decreased significantly at the 3-month (m = 20.28, m = 25.93; *p* = 0.023) and 1-year (m = 19.78, m = 24.83; *p* = 0.041) follow-up in the intervention group but not in the control group (Intervention*time: F (2, 132.595) = 4.126, p = .018). The effect size at both follow-ups was moderate to large (Cohen’s d = 0.77 0.69, respectively). No effect was found in MADRS. Between the 3-month and 1-year follow-ups for RTW, there was a significant change for the intervention group (*p* = 0.029), indicating an increase in full RTW.

**Conclusion:**

The ReTA model is an effective treatment for ED among healthcare professionals. Further research is needed with longer follow-up periods to see the long-term effects on ED, depression, and RTW.

**Trial Registry:**

NCT05314530.

## Introduction

Long-term sickness absence due to stress-related ill-health (SRIH) diagnoses has increased over the last decades in Sweden [1]. Statistics of Swedish workers found that occupations with a higher than average risk of sickness absence from all causes and from mental health problems, including SRIH, are healthcare professionals such as physicians and nurses [2], which is a major problem as the healthcare system is already understaffed [3]. This demands that effective rehabilitation interventions be developed and clinically evaluated to facilitate symptom relief, return to work, and increased workability. Previous research in the area underlines the need for more comprehensive studies focusing on long-term follow-up, methods, patient experiences, and the development of comprehensive, evidence-based treatment strategies for patients with sickness absence due to SRIH [4–15].

The existence and definition of SRIH diagnoses are complex and have been debated both clinically and in research [16], which may have implications for treatments and rehabilitation. Internationally, burnout is used to conceptualize an individual's response to chronic work stress that develops progressively, and that can eventually become chronic with severe symptoms [17]. The World Health Organization (WHO) states that burnout is characterized by 1) energy depletion or exhaustion, 2) increased mental distance from one’s job, and 3) reduced professional efficacy [18] (ICD‐11 code QD85). Research has pointed out that the WHO definition lacks the loss of cognitive functions [16], which is found to be an important diagnostic criterion in burnout. Schaufeli et al. [19], therefore, conceptualized burnout as “*a work-related state of exhaustion that occurs among employees, which is characterized by extreme tiredness, reduced ability to regulate cognitive and emotional processes and mental distancing*” [19]. In Sweden, diagnostic criteria exist for clinical burnout, i.e., exhaustion disorders (ED), and is included in the Swedish version of the International Classifications of Diseases, 10th edition (ICD‐10 code F43.8A)[20]. ED is a relatively new diagnosis in Sweden and has not been included in any ICD version outside of Sweden except for the Netherlands [21, 22]. Similarly to the conceptualization of burnout by Schaufeli et al. [19], ED is a result of prolonged, unrelieved, intensive stress without sufficient recovery, leading to a state of exhaustion that is characterized by profound fatigue, emotional instability, impaired sleep, and cognitive problems such as attentional and executive dysfunction [4] and acknowledges stressors from both work and private life [5]. Emotional and cognitive impairment are often considered core dimensions of burnout or ED due to reduced functional capacity in regulating emotions and cognitive processes [4]. For many individuals diagnosed with ED, it often requires long-term sickness absence due to extensive rest symptoms that affect their cognitive functioning [4, 23], and cognitive impairment often persists over a significant period after being diagnosed with ED [5, 15, 24], sometimes resulting in struggles to return to work and relapses into full-time sickness absence [25].

Meanwhile, clinicians and scholars argue that burnout does not exist and that burnout and ED are aspects of depression [21, 26–30]. While burnout and depression exhibit similarities, such as the experience of decreased energy, numerous researchers argue for a distinction between burnout and depression [31, 32] as separate concepts and that emotional exhaustion, a component of burnout, does not correlate with depression [26]. For instance, a recent study found that burnout complaints predicted subsequent sickness absence while depression did not [33]. A meta-analysis by Koutsimani et al. [26] also showed that there was no conclusive overlap between burnout and depression, which indicates that they are different and robust constructs. Nevertheless, individuals experiencing ED may display behaviors and characteristics reminiscent of depression or anxiety. For example, Koutsimani et al. [26] describe how employees who have been diagnosed with a depressive and/or anxiety disorder might also suffer from burnout [26]. Therefore, there is a complexity in diagnosing ED correctly due to the lack of a clear and common understanding of the association between burnout and depression [13, 26]. This is further complicated by the fact that diagnostic codes for SRIH are not consistent in Sweden, and individuals may also, due to differences in prescribing sick leave between healthcare providers, be diagnosed with, e.g., adjustment disorder or acute stress reactions and sometimes depression [26].

Leaning on the arguments that ED and depression are distinct diagnoses [26, 32], rehabilitation interventions must be tailored to the symptoms and disease expressions of ED when the aim is to reduce ED symptoms. Therefore, this study seeks to evaluate whether a rehabilitation intervention designed to target ED symptoms reduces symptoms of ED compared to depressive symptoms and contributes to return to work. In recent years, scientific projects have been conducted to develop and test rehabilitation interventions for people with long-term sickness absences due to SRIH. While most treatments tend to lead to symptom relief, there is yet no consensus on the types of rehabilitation [4, 22]. A new research book on ED [4] found some common aspects that may be successful in relation to ED. Multimodal treatment, where the intervention is directed toward several aspects of the experienced symptoms with the help of therapists from different professions, is recommended [4]. A balance between recovery, physical activity, improvement of sleep quality, and workplace involvement appears to be a meaningful factor in ED rehabilitation [4, 9, 34, 35]. Research has shown good results for combined interventions, including both the individual and organization, which led to improved symptoms of ED [10], as well as individual-focused interventions such as mindfulness, stress management, and small group discussions [35–37]. Based on previous knowledge [4–15], we developed a rehabilitation intervention tailored to ED, the Rehabilitation to Work (ReTA: Rehabilitering Till Arbete) model. This study aims to assess the impact of the ReTA model on symptoms of ED and depression and return to work.

## Methods

This study used an RCT design to evaluate if a rehabilitation intervention targeting ED is more effective for reducing symptoms of ED than depression among healthcare workers who were on LTSA for SRIH. For more detailed information about the ReTA model and details on the content of the rehabilitation intervention, see Appendix 1. We used the CONSORT checklist when writing our report [38].

### The Rehabilitation Intervention

ReTA is a multimodal rehabilitation intervention aimed at reducing symptoms of SRIH, implemented in a residential format, where participants stay on-site for three weeks and participate in physical activity, meditation, mindfulness, collegial discussions and peer support, and individual sessions with a therapist. Activities were scheduled between 8.00 and 17.00 every weekday, with the weekends free to explore the surroundings or rest. From 2016 to 2017, the ReTA model was tested among teachers using a randomized control trial (RCT) design [34]. After one year, the study showed a 16% higher rate of return to work for the intervention group. A 3-year follow-up showed that teachers participating in the intervention group were more likely to return to full-time work (S2020/95822/FS). Research shows that using interventions with proven efficacy in new contexts can be more effective than developing new interventions [39], and because of its effectiveness among teachers, ReTA was adapted to nurses and physicians who are on LTSA for SRIH.

## Participants

An external power calculation was conducted based on results from an earlier trial period of the ReTA model in a different setting [34]. It used expected changes in KEDS scores and return-to-work outcomes and showed that about 30 participants would be needed. The detailed documentation of this calculation is unavailable because it was performed by an outside collaborator.

We recruited nurses and physicians who were on long-term sickness absences due to SRIH from a national insurance database held by an insurance company owned by Sweden's labor market parties. Individuals with a medically certified sickness absence exceeding 90 days can apply for an insurance claim. All nurses and physicians who had submitted a claim with ICD codes F32 (depressive episode), F41 (anxiety syndromes), or F43 (reactions to severe stress and adjustment disorders) received a postal letter from the insurance company with information about the study and the ReTA model. They were also asked to fill in and return (in a pre-stamped envelope) permission for the insurance company to provide contact information to the project management.

The nurses and physicians who returned their permission to contact them were sent an email inviting them to complete a questionnaire consisting of demographic questions, self-reported ED, depression and general health, and occupational information. The questionnaire ended with further information about the ReTA model and a question about whether the individual was interested in participating in the rehabilitation intervention.

The eligible criteria to participate in the RCT were a score on the Karolinska Exhaustion Disorder Scale (KEDS) [40] of 19 and above assuring ED symptoms, a score on the Montgomery Åsberg Depression Rating Scale (MADRS) [41] below 34, no chronic disease or other disease that could explain the ill-health, be on sickness absence and be below 61 years. Exclusions were made for participants with a score lower than 19 in KEDS and over 34 in MADRS, who were older than 60 years, not on sickness absence at the time, and participants who reported other illnesses as the main cause of their sickness absence.

Those who responded positively to participate in the study and met the inclusion criteria were listed and randomly assigned to the intervention group or the control group by selecting every third responder. People in the project group conducted randomization and contacted participants. If the participant, when contacted, declined participation, the next third person on the list was contacted.

The control group did not receive any treatment from the research group. They received the care offered in healthcare or occupational health services, and the type of help they received can vary depending on where in the country they lived, because there are regional differences in diagnosing and prescribing sickness absence [42] and rehabilitation.

Due to the lack of consensus among healthcare providers on how to diagnose ED, we used the ICD-10 codes F32, F41, and F43 in order not to miss people who may be suffering from ED but have been misdiagnosed with anxiety or depression. We used cut-off in KEDS and MADRS to ensure that those included in the study were showing definite symptoms of ED but not symptoms of severe depression. In line with Besèr et al. [40], we used a cut-off at 19 points to indicate symptoms of ED for inclusion in the study.

In total, 356 received an email invitation to the questionnaire, and 294 answered. Of those, 113 were eligible participants (181 did not meet the inclusion criteria or did not answer the follow-up questionnaires). 72 answered both the 3-month follow-up and the 1-year follow-up (intervention group: n = 30, 42%, and control group: n = 42, 58%). Only those who completed all three questionnaires were included in the analyses (The participant flow diagram is presented in Appendix 2).

## Data Collection

The intervention was carried out on two occasions: Trial 1 in May 2022 and Trial 2 in December 2022. The baseline for Trial 1 is all those who responded prior to the intervention starting in May, while the baseline for Trial 2 was all those who responded after the intervention started in May and before the intervention started in December (see Table 1 for more information on the time for data collection). Both the intervention group and the control group received an initial baseline questionnaire and were followed up with a questionnaire three months and one year after the intervention group had finished their treatment.

**Table 1:** Time points for data collection

	Baseline survey	3-month follow-up	1-year follow-up	
Trial 1	March, 2022	August, 2022	May—July, 2023	
Trial 2	October, 2022	March, 2023	November 2023- January 2024	

Study data were collected and managed using the REDCap (Research Electronic Data Capture) tool hosted at Karolinska Institutet. REDCap is a secure, web-based software platform designed to support data capture for research studies, providing: 1) an intuitive interface for validated data capture; 2) audit trails for tracking data manipulation and export procedures; 3) automated export procedures for seamless data downloads to common statistical packages; and 4) procedures for data integration and interoperability with external sources [43, 44].

## Measurements

### Exhaustion Disorder

The degree of self-reported ED was measured using KEDS, comprising nine items based on nine symptoms of exhaustion (sleep, concentration, memory, physical exhaustion, endurance, recovery, sensitivity to sensations, perceptions of demands, and anger/irritation) with the response alternatives ranging from 0 to 6 [40]. The overall KEDS score was calculated by summing the nine items into an index ranging from 0 to 54, with a higher value indicating more severe symptoms. In previous assessments, KEDS has shown good validity and satisfactory internal consistency [9].

### Depression

The degree of self-reported depression was measured using MADRS, which comprises eight items based on symptoms of depression (sleep, concentration, appetite, disposition, feelings of anxiety, initiative abilities, emotional involvement, and pessimism), with the response alternatives ranging from 0 to 6 [41]. The overall MADRS score was calculated by summing the eight items to an index ranging from 0 to 48, with a higher value indicating more severe symptoms. In previous assessments, MADRS has shown good validity and acceptable inter-rater reliability [45].

### Return to Work

Respondents were asked to what degree (percentages) they were on sick leave, with five response categories: 100%, 75%, 50%, 25%, and 0%, responding to the levels of sickness benefits granted by the Swedish Social Insurance Agency [46]. Categories were recorded into three categories: Full-time sick absence (100% to 75%), part-time sickness absence (50%-25%), and returned to work (0%).

## Analytical Strategy

Statistical tests were conducted to investigate the potential effect of the intervention on the level of self-reported ED symptoms (KEDS) and depressive symptoms (MADRS). In the analyses, the intervention and control groups from Trials 1 and 2 were combined into one total intervention group and one total control group. A Shapiro–Wilk test showed that the data were normally distributed (p = 0.6), which was why a parametrical test was used.

To assess differences in mean values of KEDS and MADRS for the intervention and control groups over time, we used a Linear Mixed Model (LMM) with fixed effects for intervention and control groups, adjusting for the time-invariant variables age, gender, profession, and trial group. An AR1 covariance structure was used for repeated measures over time. The model was fitted using Restricted Maximum Likelihood (REML), and model fit was assessed using Akaike's Information Criterion (AIC). Analysis was carried out with SPSS version 28. The Type III fixed effects are presented with degrees of freedom (df), F- and p-values. Next, mean scores for KEDS and MADRS at baseline, 3-month follow-up and 1-year follow-up are presented along with standard error (Std. E.), 95% confidence interval (CI) and within-group Δ change. Effect sizes (Cohen’s d) were calculated as the between-group difference in estimated marginal means divided by the residual standard deviation from the mixed-model (square root of the AR(1) diagonal term).

Descriptive statistics show the distribution at 3-month and 1-year follow-ups for the intervention and control groups, respectively, to assess the degree of return to work. Chi-square was used to test differences between and within groups.

## Ethics Approval

The present project has been reviewed and approved by the Swedish ethical review authority (2021–06571-01; 2022–03866-02). The project leaders have implemented the ReTA model before, and with this experience, we were been able to anticipate any risks and address them. Björn Löfman, a licensed psychologist and part of the project management team, was available to the participants during the intervention.

## Results

### Sample Description

In the study sample of 72 individuals (Table 2), the mean age was 44.9 years (SD = 8.9) and varied from 26 to 60 years. A small share were men (n = 5; 6.9%), and 67 (9.13%) were female. The study included 22 (31%) physicians and 50 (69%) nurses. Furthermore, 58 (80.6%) reported having a partner, and 53 (73.6%) reported living with their partner. 41 (56.9%) also reported having children living at home.

**Table 2:** Descriptives of study participants

	Total n(%)	Intervention n	Control n	*P*-value
Profession				0.120
Nurses	50 (69.4%)	18	32	
Physicians	22 (30.6%)	12	10	
Gender				0.938
Women	67 (93.1%)	28	39	
Men	5 (6.9%)	2	3	
Trial group				0.102
Trial 1	28 (38.9%)	15	13	
Trial 2	44 (61.1%)	15	29	
Age (m (SD))	44.65 (8.9)	43.43 (8.3)	45.49 (9.3)	0.467

### Effect on KEDS and MADRS

The result from Table 3 shows no statistical significance of the main effect from the intervention (F(1, 37.912) = 3.364, p = 0.074) but a significant effect from time (F(2, 132.595) = 33.812, p < 0.001) on KEDS scores. Importantly, the results were statistically significant for the interaction between intervention and time (F(2, 132.595) = 4.126, p = 0.018), indicating that the intervention had a greater impact over time (intervention group) than conventional rehabilitation (control group). The AR1 diagonal parameter indicates statistically significant within-subject variability in KEDS scores over time (Wald Z = 5.73, *p* =  < 0.001).

**Table 3:** Type III Tests of Fixed Effects adjusted for age, gender, profession, and trial group

			KEDS						MADRS				
	df numerator		df denominator		F		*p*-value		df denominator		F		p-value
Intercept	1		37.836		161.350		< 0.001		68.776		559.547		< 0.001
Intervention group	1		37.912		3.364		0.074		68.776		0.320		0.573
Time	2		132.595		33.812		< 0.001		94.217		18.799		< 0.001
Intervention * Time	2		132.595		4.126		0.018		94.217		2.610		0.079
AIC			1355.44						1327.96				

For MADRS scores, no statistically significant type III fixed effects were observed, except for time (p < 0.001), suggesting a general reduction in depressive symptoms over time in both groups. The AR1 parameter was also statistically significant (Wald Z = 2.51, p = 0.012), indicating meaningful within-subject correlation over time in MADRS scores.

Table 4 displays how a pairwise comparison at each time point illustrates the change in score over time for the intervention and control groups. At baseline (Table 4), there were no statistically significant differences between the intervention and control groups in mean KEDS values (I: m = 27.95 and C: m = 29.79, *p* = 0.523). At 3-month follow-up, the mean KEDS value decreased statistically significantly (*p* = 0.023) more in the intervention group (m = 20.28) compared to the control group (m = 25.93), with a moderate to large effect size (Cohen’s d = 0.77). Similarly, at the one-year follow-up, the mean KEDS score decreased statistically significantly (*p* = 0.041) more in the intervention group (m = 19.78) than in the control group (m = 24.83), with a moderate to large effect size (Cohen’s d = 0.69). Notably, the intervention group’s mean KEDS score approached the clinical cut-off (> 19) for ED [40], suggesting a clinically meaningful improvement.

**Table 4:** Mean scores for KEDS and MADRS for intervention and control groups at baseline and follow-ups

	KEDS (0–30)				MADRS (0–48)			
	Intervention	Control	Between-group difference in change	*p*-value	Intervention	Control	Between-group difference in change	*P*-value
Baseline m (SE)	27.95 (2.30)	29.49 (2.36)	1.55 (2.41)	0.523	18.36 (2.13)	18.57 (2.19)	0.211	0.925
95% CI	23.31–32.58	24.73–34.26	-3.29–6.38		14.07–22.66	14.16–22.99	-4.69–4.27	
3-month follow-up								
m (SE)	20.28 (2.30)	25.93 (2.36)	1.56 (2.41)	0.023	12.83 (2.13)	16.16 (2.19)	3.33	0.141
95% CI	15.64–24.92	21.17–30.70	0.82–10.49		8.53–17.13	11.74–20.58	-1.15–7.81	
Δ-change vs baseline	-7.67	-3.56	-4.11 (95% CI: -7.8- -0.4)		-5.53	-2.41	-3.12 (95% CI: -7.8 – 1.1)	
Effect size			0.77	0.023			0.58	0.141
1-year follow-up								
m (SE)	19.78 (2.30)	24.83 (2.36)	5.05 (2.41)	0.041	12.96 (2.13)	15.45 (2.19)	2.49	0.269
95% CI	15.14–24.42	20.07–29.60	0.22–9.89		8.67–17.26	11.03–19.87	-1.99–6.97	
Δ-change vs baseline	-8.17	-4.66	-3.51 (95% CI:** -**6.9- -0.2)		-5.40	-3.12	-2.3 (95% CI: -7.0 – 2.0)	
Effect size			0.69	0.041			0.43	0.269

^a^Clinical cut-off is 19

^b^Clinical cut-off is 34

The results for MADRS confirm the type II test, showing no statistically significant pairwise differences at any time point nor delta changes. The effect size was small to moderate (Cohen’s d = 0.58 and 0.43, respectively).

### Return to Work

At baseline, all participants were either on part-time or full-time sickness absence, with no statistically significant difference between groups (*p* = 0.253; Table 5). Across all three measure points, there was a significant change in return to work for both groups (*p* =  < 0.001). At 3-month follow-up, 39.0% of participants in the control group and 23.3% of participants in the intervention group had returned to work full-time (*p* = 0.017). Meanwhile, a larger number of participants in the intervention group were still on full-time sickness absence (36.7% and 9.8%).

**Table 5:** Descriptives of the degree of sickness absence and return to work for the intervention and control groups at baseline, 3-month and 1-year follow-up

		Full-time sickness absence	Part-time sickness absence	Returned to work full-time	P-value for group differences
Baseline	Intervention	14 (46.7%)	16 (53.3%)	0 (0.0%)	0.253
	Control	14 (33.3%)	28 (66.7%)	0 (0.0%)	
3-month follow-up	Intervention	11 (36.7%)	12 (40.0%)	7 (23.3%)	0.017
	Control	4 (9.8%)	21 (51.2%)	16 (39.0%)	
1-year follow-up	Intervention	7 (23.3%)	6 (20.0%)	17 (56.7%)	0.629
	Control	7 (17.1%)	12 (29.3%)	22 (53.7%)	

Between the 3-month and 1-year follow-up, the intervention group experienced within-group changes (Pearson Chi-square *p* = 0.029), indicating a significant increase in return to work full-time. No within-group changes existed in the control group (Pearson Chi-square *p* = 0.121). At the one-year follow-up, the number of participants with full-time sickness absence in the control group increased to seven, while it decreased in the intervention group (from 11 to 7). Meanwhile, the percentage that reported being back full-time at work increased 143% in the intervention group and 38% in the control group.

## Discussion

In this study, we evaluated the ReTA model, a rehabilitation intervention designed to target symptoms of ED. ReTA is a multimodal rehabilitation intervention in residence format, including peer support and individual-focused activities for nurses and physicians in long-term sickness absence for SRIH. Using an RCT design, this study shows that the ReTA model had a measurable impact on the mean value of KEDS (symptoms of ED) but not MADRS (depressive symptoms) and seems to facilitate return to work. The study contributes to the current lack of clinically evaluated rehabilitation interventions [47] in reducing symptoms of primarily ED and not depression. Overall, the results show that the ReTA model is a suitable intervention to reduce symptoms of ED.

At 1-year follow-up, the KEDS score indicated that the level of ED symptoms continued to decrease in both groups, but to a significantly larger degree in the intervention group. These results are in contrast with previous research where the differences between the treatment and control groups did not persist after a 1-year follow-up [4, 6, 12]. Nurses and physicians in the control group likely received some rehabilitation activities between the 3-month and 1-year follow-up. While many of the conventional rehabilitation interventions offered usually result in symptom relief [11, 48], they seem to be less effective than the ReTA model. At the 1-year follow-up, the mean KEDS value for the intervention group was reduced to 19, which is the clinical cut-off for ED.

Assessing the return to work by measuring the percentage of sickness absence is complex. The extent and reasons for continued sickness absence are influenced by various external factors, including national social insurance regulations, the duration of the individual's sickness absence, and the physician’s experience with and attitude toward SRIH and ED [49]. For example, an individual’s sickness benefit period might end, requiring them to return to work even if they are not yet ready for it. In Sweden, after 90 days of sickness absences, individuals are assessed to determine if they can perform other work at their employer. After 180 days, they are assessed against any work on the labor market. At the 3-month follow-up, we expected that the control group would have returned to work to a greater extent, which was confirmed in the data. This was because the intervention participants remained on sickness absence from the time they learned they were going to participate in the intervention and throughout the three-week ReTA residence. They could continue to be on sickness absence benefits during this period since they were going through treatment. Meanwhile, the individuals in the control group probably returned to work as usual, following the guidelines for sickness absence benefit. Research indicates that residual symptoms of ED may create a vulnerability, risking a relapse into sickness absence when exposed to increased work stress upon returning to work [15]. This is particularly relevant for healthcare workers, as the working conditions in the healthcare sector are extremely strained, with many physicians and nurses reporting significant levels of burnout [50–52]. Therefore, recurring full-time sickness absences must be acknowledged when considering returning to work. At the 1-year follow-up, we observed a tendency for individuals in the control group to return to full-time sickness absence. In contrast, the intervention group showed a positive trend toward returning to work. This indicates that the ReTA model may be valuable for a more sustainable return to work. However, longer follow-up periods are necessary to see any long-term effects of the ReTA model compared to traditional rehabilitation offered and whether the ReTA gives the participants tools to handle stress when returning to work compared to the controls. Therefore, it is important that any rehabilitation intervention not only reduces symptoms but also provides participants with tools and resources for a sustainable return to work.

Multimodal rehabilitation has received criticism as it tends to lead to longer sickness absence [49]. One reason multimodal rehabilitation takes longer is that it demands coordination between several professions. In the ReTA model, we used a multimodal approach in a compressed time in a residential format for three weeks. The results from our study are consistent with previous research describing the balance between recovery, physical activity, sleep intervention, and workplace interventions as meaningful in the treatment of ED [4]. Although sickness absence due to SRIH is a common problem, few clinically evaluated treatments or rehabilitation interventions exist [4–15]. We found no evidence that the intervention reduced symptoms of depression. This further supports the evidence that ED or burnout and depression are different constructs that need different treatments aimed at the specific and different symptoms of the conditions. This study, therefore, makes a valuable empirical contribution to the research field as we found that the ReTA model seems to be effective in treating ED primarily and not other mental health conditions, such as depression. The results from this study, as well as the results from the study of the ReTA model among teachers [34], indicate that the ReTA model could be an effective treatment for ED primarily and among different occupational groups. It would be of great value to evaluate the ReTA model in additional occupational groups to establish these findings. Also, we agree with previous research that expresses the need for studies focusing on long-term follow-up, methods, patient experiences, and the development of comprehensive, evidence-based treatment strategies for individuals on sickness absence for ED [4–15] to better understand how to treat ED specifically.

### Strengths and Limitations

This study has both strengths and limitations, which we discuss here. The strength of the study is the use of an RCT design to test the effect of a rehabilitation intervention on ED symptoms and return to work. Previous research has concluded that more comprehensive RCT studies with an acceptable control group are needed to draw more reliable conclusions on the effectiveness of rehabilitation interventions [35, 53–55]. However, a limitation is that we don’t know whether the control group has been offered any treatment and what this treatment included; therefore, we included a larger control group. Furthermore, the follow-up time is limited to one year, which can be considered short and requires further follow-up.

Although we had no loss of follow-up in the intervention group, 72 out of the 114 participants in the control group did not answer both follow-up surveys and were excluded from the analyses. A larger share of drop-out is expected in the control group, as their motivation to follow through may be less, as they have not received any treatment. There could also be individual factors such as general health status, life situations, medical treatments, and work environment conditions that cannot be controlled during the follow-up period and could affect the results. Therefore, further research and controlling for relevant underlying factors are needed to establish the causal effect of the intervention on ED and ensure that the intervention is most effective for reducing symptoms of ED and not depression.

Although a power calculation was performed before starting the study based on data from a previous ReTA model trial [34], the detailed documentation of this calculation is unavailable. This reduces the transparency of the study design and the clarity of the findings. Future studies with fully documented sample size calculations and pre-defined primary outcomes are needed to verify these exploratory results.

Fewer men answered the questionnaires and wanted to participate in the study than women. There could be many reasons for this. For instance, more women than men work in the health sector [56], and more women than men are on sickness absence due to SRIH [2]. More men would have been needed in the sample to look at gender differences and determine whether the results would differ depending on gender. However, a study by Glise et al. [54] did not find a statistically significant difference between women and men regarding the level of ED, and women and men who seek care for SRIH do not seem to differ substantially regarding the burden of symptoms [54]. This indicates that the unbalanced distribution of men and women in the study might not influence the results.

Although all insured individuals can make an insurance claim and thus be included in the database, not everyone does this. We do not know if all individuals received the postal letter with the first information or the e-mail with the link to the questionnaire. Also, between claiming insurance, being contacted by the insurance company, answering the baseline questionnaire, and randomizing participants, some nurses and physicians initiated returning to work. Some had even returned to work but were back on sickness absence. However, based on KEDS values, we could see that all participants had significant symptoms of ED.

Lastly, although clinical diagnosis descriptions for ED have been established in Sweden (ICD-code F43.8), the ICD code noted in the medical certificate varies, i.e., individuals might have an alternative ICD code in the medical certificate. We, therefore, chose to extract all nurses and physicians with an insurance claim of ICD codes F32, F41, and F43 from the national insurance database and restrict inclusion criteria in relation to the level of ED and depression, which we believe is a strength, as individuals otherwise could have been missed due to misdiagnosis. The development of rehabilitation interventions for treating symptoms of ED should continue. We agree with previous research that it would be beneficial for research on rehabilitation interventions if the assessment of ED relied on agreed-upon guidelines [5, 6], and it would also be effective if this definition could be internationally agreed upon, to ensure that interventions aimed at treating ED are effective primarily for that condition.

## Conclusion

This study indicates that the ReTA model for rehabilitation effectively reduces ED symptoms but not depressive symptoms for nurses and physicians in sickness absence for SRIH. There was a tendency for the model to contribute to a more sustainable return to work for the intervention group, but this needs further research. There is a need for tailored rehabilitation interventions for ED that facilitate symptom relief and sustainable return to work.

## Data Availability

The raw ReTA data is protected and unavailable due to data privacy laws. All collected data is stored on secure servers and will only be available to researchers in the project. The material is processed on a special confidential computer server at Karolinska Institutet. We can distribute aggregated data on request.

## Description of the model: Rehabilitation to Work (ReTA)

The ReTA treatment is a three-week multimodal rehabilitation program in a residential setting. The rehabilitation, such as lectures, exercise, and therapy, takes place during the weekdays from 8:00 to 17:00, and participants stay for three weeks to allow for reflection and distance from the stressors at home. The residential part takes place at Rygginstitutet (The Back Institute) outside Stockholm. Rygginstitutet has full accommodation facilities for participants, with single rooms with private bathrooms. There are common areas for socializing and kitchenettes. They also have a swimming pool, gym, exercise areas, walking tracks, and rooms for mindfulness practice and various intervention activities. Rygginstitutet is located in a nice green area, right next to the ocean, with easy access to Stockholm. Half of the time is devoted to physical exercise such as powerwalking with poles and water gymnastics with physiotherapists, as well as to mindfulness and yoga. Participants complete between 2–3 sessions of mixed physical activity each day, each session being between 30 min and 1.5 h long. A fitness test is also carried out at the beginning and end of the intervention to compare whether fitness levels have improved during the intervention.

The second part is devoted to lectures and discussion groups on topics relevant to burnout and stress-related ill-health. Through the lectures, we try to give the participants more knowledge about symptoms and symptom development in burnout, recovery, and factors at work that can contribute to stress. The lectures are once a week and vary between 1 and 2 h. A new feature of the treatment, which differs from previous treatments, is that the rehabilitation group consists of people with the same professional background. The groups also conducted peer discussions, which were conducted among the doctors and the nurses separately. There is research evidence showing that peer support from others who are in the same situation seems to be advantageous in rehabilitation and recovery from burnout [57]. The peer discussions had different predetermined themes, and the themes were based on the results of the initial questionnaire and were about different stressors in work, leisure, and family life. The meaning of the peer discussions is that the participants advise each other about different shortcomings and problems in work and life. Participants also have individual discussions with a psychologist to clarify issues that need to be processed and perhaps changed in the different aspects of life. The participants conduct the peer counseling groups about 4 times a week and the length of the sessions varies between 1 and 2 h. The number of individual sessions with a psychologist can depend on the needs of the individual. During the last days of the residential treatment, each participant writes an action plan, which should contain three things that they want to continue working on when they return home. After one month, there is a follow-up interview for research purposes, and at 3 months, there is a check-up by the psychologists where the action plan is discussed, and after one year, there is another follow-up interview for research purposes. Participants also receive a follow-up questionnaire at 3 months and 1 year to monitor KEDS, MADRS, and return to work.

The control group consists of the same number of participants as the intervention group, and they have not received any treatment from the research group. They receive the care offered in their region, and the type of help they receive can vary depending on where in the country they live because, as mentioned, there are regional differences in diagnosing and prescribing sickness absence [42]. The control group, like the intervention group, receives an initial questionnaire and is also followed up with a questionnaire 3 months and 1 year after the intervention group has finished their treatment. This means that it is difficult to know whether the control group has been offered any kind of treatment during this period or whether they completed treatment before the study was carried out. However, in the follow-up surveys, we ask the respondents to list the interventions they have received in the last months, and the answers can, therefore, indicate whether the respondents have received any intervention.

For questions or more information on the treatment, please contact: reta@kids.ki.se.

## References

[1] The Swedish Social Insurance Agency. Follow-up of the development of sickness absence in 2020 [uppföljning av sjukfrånvarons utveckling 2020].

[2] Lidwall U, Bill S, Palmer E, Olsson BC. Mental disorder sick leave in Sweden: a population study. Work. 2018;59(2):259–72. 10.3233/WOR-172672 29355123

[3] Olsson, Å. Continued shortage of licensed health and medical care personnel [Fortsatt brist på legitimerad hälso- och sjukvårdspersonal]. The Swedish National Board of Health and Welfare 2023.

[4] Åsberg M, Blomberg C, Brändström L, Clason van de Leur J, Eskilsson T, Finnes A, et al. Exhaustion disorders [Utmattningssyndrom]. 1st ed. Stockholm: Gothia kompetens; 2024. 304 p.

[5] Ellbin S, Jonsdottir IH, Bååthe F. “Who I am now, Is more me.” An interview study of patients’ reflections 10 years after exhaustion disorder. Front Psychol. 2021 Dec 9;12:752707. 10.3389/fpsyg.2021.752707 PMC8699002 34955973

[6] Ahola K, Toppinen-Tanner S, Seppänen J. Interventions to alleviate burnout symptoms and to support return to work among employees with burnout: systematic review and meta-analysis. Burnout Res. 2017;4:1–11.

[7] Soegaard HJ. Variation in effect of intervention studies in research on sickness absence. Open Access J Clin Trials. 2012;4:1–20.

[8] Karlson B, Jönsson P, Österberg K. Long-term stability of return to work after a workplace-oriented intervention for patients on sick leave for burnout. BMC Public Health. 2014;14(1):821. 10.1186/1471-2458-14-821 PMC4137088 25106094

[9] van de Leur JC, Buhrman M, Wallby K, Karlström A, Johansson F. Associations between improvements in psychological variables and subsequent sick leave among persons receiving a multimodal intervention for exhaustion disorder. BMC Public Health. 2023;11(23):1976. 10.1186/s12889-023-16799-x PMC10568869 37821913

[10] Pijpker R, Vaandrager L, Veen EJ, Koelen MA. Combined interventions to reduce burnout complaints and promote return to work: a systematic review of effectiveness and mediators of change. Int J Environ Res Public Health. 2020;17(1):55. 10.3390/ijerph17010055 PMC6981402 31861699

[11] Perski O, Grossi G, Perski A, Niemi M. A systematic review and meta-analysis of tertiary interventions in clinical burnout. Scand J Psychol. 2017;58(6):551–61. 10.1111/sjop.12398 29105127

[12] Lindegård A, Glise K, Wiegner L, Reinhardt P, Ellin S, Pettersson S, et al. Effects of additional individually tailored interventions on sick-leave and symptoms in patients with exhaustion disorder: a randomized controlled trial. J Rehabil Med. 2022;24(54):2941. 10.2340/jrm.v54.2941 PMC9422869 35801865

[13] Lambreghts C, Vandenbroeck S, Goorts K, Godderis L. Return-to-work interventions for sick-listed employees with burnout: a systematic review. Occup Environ Med. 2023;80(9):538–44. 10.1136/oemed-2023-108867 PMC10447379 37500536

[14] Kärkkäinen R, Saaranen T, Hiltunen S, Ryynänen OP, Räsänen K. Systematic review: factors associated with return to work in burnout. Occup Med. 2017;67(6):461–8. 10.1093/occmed/kqx093 28898968

[15] Glise K, Wiegner L, Jonsdottir IH. Long-term follow-up of residual symptoms in patients treated for stress-related exhaustion. BMC Psychol. 2020;19(8):26. 10.1186/s40359-020-0395-8 PMC7081527 32188513

[16] Bianchi R, Schonfeld IS. Burnout: half a century of controversy. Occup Med. 2024;74(6):400–2. 10.1093/occmed/kqae052 39313245

[17] Edú-Valsania S, Laguía A, Moriano JA. Burnout: a review of theory and measurement. Int J Environ Res Public Health. 2022;19(3):1780. 10.3390/ijerph19031780 PMC8834764 35162802

[18] World Health Organization. Burn-out an “occupational phenomenon”: International Classification of Diseases [Internet]. 2019 [cited 2024 May 24]. Available from: https://www.who.int/news/item/28-05-2019-burn-out-an-occupational-phenomenon-international-classification-of-diseases

[19] Schaufeli WB, Desart S, De Witte H. Burnout Assessment Tool (BAT)—development, validity, and reliability. Int J Environ Res Public Health. 2020;17(24):9495. 10.3390/ijerph17249495 PMC7766078 33352940

[20] Reinhardt P, Ellbin S, Carlander A, Hadzibajramovic E, Jonsdottir IH, Lindqvist Bagge A. Is the road to burnout paved with perfectionism? The prevalence of obsessive-compulsive personality disorder in a clinical longitudinal sample of female patients with stress-related exhaustion. J Clin Psychol. 2024;80(2):391–405. 10.1002/jclp.23617 37866970

[21] Lindsäter E, Svärdman F, Wallert J, Ivanova E, Söderholm A, Fondberg R, et al. Exhaustion disorder: scoping review of research on a recently introduced stress-related diagnosis. BJPsych Open. 2022;8(5):e159. 10.1192/bjo.2022.559 PMC9438479 36458830

[22] Van Dam A. A clinical perspective on burnout: diagnosis, classification, and treatment of clinical burnout. Eur J Work Organ Psychol. 2021;30(5):732–41.

[23] The Swedish Social Insurance Agency. Sickness absence due to psychiatric diagnoses Analysis and forecast. [Sjukfrånvaro i psykiatriska diagnoser. Analys och prognos]. Report No.: 2020:8. The Swedish Social Insurance Agency. 2020.

[24] Jonsdottir IH, Nordlund A, Ellbin S, Ljung T, Glise K, Währborg P, et al. Working memory and attention are still impaired after three years in patients with stress-related exhaustion. Scand J Psychol. 2017;58(6):504–9. 10.1111/sjop.12394 29023756

[25] Toropova A, Björk Brämberg E, Bergström G. Return to work trajectories of Swedish employees on sick-leave due to common mental disorders. J occup rehabil. 2025;35(3):479–90. 10.1007/s10926-024-10216-9. PMC12361341 38907784

[26] Koutsimani P, Montgomery A, Georganta K. The relationship between burnout, depression, and anxiety: a systematic review and meta-analysis. Front Psychol. 2019;13(10):284. 10.3389/fpsyg.2019.00284 PMC6424886 30918490

[27] Schonfeld IS, Bianchi R. Burnout and depression: two entities or one? J Clin Psychol. 2016;72(1):22–37. 10.1002/jclp.22229 26451877

[28] Bianchi R, Schonfeld IS, Laurent E. Burnout–depression overlap: a review. Clin Psychol Rev. 2015;36:28–41. 10.1016/j.cpr.2015.01.004 25638755

[29] Bianchi R, Schonfeld IS, Laurent E. Is burnout a depressive disorder? A reexamination with special focus on atypical depression. Int J Stress Manag. 2014;21(4):307–24.

[30] Bakker AB, Schaufeli WB, Demerouti E, Janssen PPM, Van Der Hulst R, Brouwer J. Using equity theory to examine the difference between burnout and depression. Anxiety Stress Coping. 2000;13(3):247–68.

[31] De Beer LT, Schaufeli WB. Burnout reflections: musings on Bianchi and Schonfeld’s five focal areas. Occup Med. 2025;75(1):79–80. 10.1093/occmed/kqae134 40190122

[32] Kainalainen A, Korhonen P, Penttinen MA, Liira J. Job stress and burnout among Finnish municipal employees without depression or anxiety. Occup Med. 2024;74(3):235–41. 10.1093/occmed/kqae019 38661817

[33] Brulin E, Wilczek A, Ekberg K, Lidwall U, De Beer LT, Hadzibajramovic E, et al. Predictive value of burnout complaints and depressive symptoms for medically certified sickness absence among physicians in Sweden: a 1 year follow-up observational study. BMJ Open. 2025;15(4):e090966. 10.1136/bmjopen-2024-090966 PMC11987104 40216422

[34] Nygren Å, Löfman B, Näsman P, Ekerlund L, Kjellström B. Residential treatment helps teachers with exhaustion disorder return to work [Internatbehandling hjälper lärare med utmattningssyndrom tillbaka iarbete.] Läkartidningen. 2019 May 17; Available from: https://lakartidningen.se/klinik-ochvetenskap-1/artiklar-1/originalstudie/2019/05/internatbehandling-hjalper-larare-med-utmattningssyndrom-tillbaka-i-arbete/31192423

[35] Van De Leur JC, Buhrman M, Åhs F, Rozental A, Jansen GB. Standardized multimodal intervention for stress-induced exhaustion disorder: an open trial in a clinical setting. BMC Psychiatry. 2020;20(1):526. 10.1186/s12888-020-02907-3 PMC7643309 33153461

[36] Peterson U, Bergström G, Samuelsson M, Åsberg M, Nygren Å. Reflecting peer‐support groups in the prevention of stress and burnout: randomized controlled trial. J Adv Nurs. 2008;63(5):506–16. 10.1111/j.1365-2648.2008.04743.x 18727753

[37] Tamminga SJ, Emal LM, Boschman JS, Levasseur A, Thota A, Ruotsalainen JH, et al. Individual-level interventions for reducing occupational stress in healthcare workers. The Cochrane Database of Syst Rev. 2023;2023(5):CD002892. 10.1002/14651858.CD002892.pub6. PMC10175042 37169364

[38] Schulz KF, Altman DG, Moher D, for the CONSORT Group. CONSORT 2010 Statement: updated guidelines for reporting parallel group randomised trials. BMJ. 2010 Mar 23;340(mar23 1):c332–c332. 10.1136/bmj.c332 PMC2844940 20332509

[39] Moore G, Campbell M, Copeland L, Craig P, Movsisyan A, Hoddinott P, et al. Adapting interventions to new contexts—the ADAPT guidance. BMJ. 2021;3:n1679. 10.1136/bmj.n1679 PMC8329746 34344699

[40] Besèr A, Sorjonen K, Wahlberg K, Peterson U, Nygren Å, Åsberg M. Construction and evaluation of a self rating scale for stress-induced exhaustion disorder, the Karolinska Exhaustion Disorder Scale. Scand J Psychol. 2014;55(1):72–82. 10.1111/sjop.12088 PMC4235404 24236500

[41] Montgomery SA, Åsberg M. A new depression scale designed to be sensitive to change. Br J Psychiatry. 1979;134(4):382–9. 10.1192/bjp.134.4.382 444788

[42] The Swedish Social Insurance Agency. Sickness absence patterns. Differences between counties, municipalities and healthcare units. [Sjukskrivningsmönster. Skillnader mellan län, kommuner och vårdenheter.]

[43] Harris PA, Taylor R, Minor BL, Elliott V, Fernandez M, O’Neal L, et al. The REDCap consortium: building an international community of software platform partners. J Biomed Inform. 2019;95:103208. 10.1016/j.jbi.2019.103208 PMC7254481 31078660

[44] Harris PA, Taylor R, Thielke R, Payne J, Gonzalez N, Conde JG. Research electronic data capture (REDCap)—a metadata-driven methodology and workflow process for providing translational research informatics support. J Biomed Inform. 2009;42(2):377–81. 10.1016/j.jbi.2008.08.010 PMC2700030 18929686

[45] Davidson J, Turnbull CD, Strickland R, Miller R, Graves K. The Montgomery-Åsberg Depression Scale: reliability and validity. Acta Psychiatr Scand. 1986;73(5):544–8. 10.1111/j.1600-0447.1986.tb02723.x 3751660

[46] The Swedish Social Insurance Agency. Sickness benefit. 2025 [cited 2025 Apr 15]. Available from: https://www.forsakringskassan.se/english/sick/employee/sickness-benefit

[47] Venning A, Oswald TK, Stevenson J, Tepper N, Azadi L, Lawn S, et al. Determining what constitutes an effective psychosocial ‘return to work’ intervention: a systematic review and narrative synthesis. BMC Public Health. 2021;21(1):2164. 10.1186/s12889-021-11898-z PMC8620530 34823501

[48] Aronsson G, Lundberg U. Rehabilitation and coordination – final report: Evaluation of the Rehsam research programme 2009–2011 [Rehabilitering och samordning – slutrapport: Utvärdering av Rehsams forskningsprogram 2009–2011]. Stockholm: Department of Psychology, Stockholm University; 2017.

[49] Swedish Government Official Reports. The right conditions for sickness certification [Rätt förutsättningar för sjukskrivning]. Stockholm; 2023. Report No.: SOU 2023:48.

[50] Brulin E, Lidwall U, Seing I, Nyberg A, Landstad B, Sjöström M, et al. Healthcare in distress: a survey of mental health problems and the role of gender among nurses and physicians in Sweden. J Affect Disord. 2023;339:104–10. 10.1016/j.jad.2023.07.042 37433382

[51] Hagqvist E, Ekberg K, Lidwall U, Nyberg A, Landstad BJ, Wilczek A, et al. The Swedish Healthphys study: study description and prevalence of clinical burnout and major depression among physicians. Chronic Stress. 2022;6:247054702210838. 10.1177/24705470221083866 PMC8984863 35402760

[52] Christiansen F, Gynning BE, Lashari A, Johansson G, Brulin E. Associations between effort–reward imbalance and risk of burnout among Swedish physicians. Occupational Medicine, 2024;74(5):355–363. 10.1093/occmed/kqae039 PMC11285157 38970463

[53] Eklund M, Erlandsson LK. Return to work outcomes of the redesigning daily occupations (ReDO) program for women with stress-related disorders—a comparative study. Women Health. 2011;51(7):676–92. 10.1080/03630242.2011.618215 22082247

[54] Glise K, Ahlborg G, Jonsdottir IH. Course of mental symptoms in patients with stress-related exhaustion: does sex or age make a difference? BMC Psychiatry. 2012;12(1):12-18. 10.1186/1471-244X-12-18 PMC3338076 22409935

[55] National Board of Health and Welfare. Exhaustion disorder: stress-related mental illness [Utmattningssyndrom: stressrelaterad psykisk ohälsa]. Stockholm: National Board of Health and Welfare; 2003.

[56] National Board of Health and Welfare. Statistics on healthcare personnel [Statistik om hälso- och sjukvårdspersonal]. National Board of Health and Welfare. 2023. Available from: https://www.socialstyrelsen.se/statistik-och-data/statistik/alla-statistikamnen/halso-och-sjukvardspersonal/

[57] Eskilsson T, Fjellman‐Wiklund A, Ek Malmer E, Stigsdotter Neely A, Malmberg Gavelin H, Slunga Järvholm L, et al. Hopeful struggling for health: experiences of participating in computerized cognitive training and aerobic training for persons with stress‐related exhaustion disorder. Scand J Psychol. 2020;61(3):361–8. 10.1111/sjop.12623 31995652

